# The complete plastome sequence of *Rumex japonicus* Houtt.: a medicinal plant

**DOI:** 10.1080/23802359.2019.1704194

**Published:** 2020-01-08

**Authors:** Gurusamy Raman, Soung Jae Cho, SeonJoo Park

**Affiliations:** a Department of Life Sciences, Yeungnam University, Gyeongsan, Republic of Korea;; b Korea Environment Assessment Group, Uiwang-si, Republic of Korea

**Keywords:** *Rumex japonicus*, chloroplast genome, medicinal plant, next-generation sequencing, Polygonaceae

## Abstract

*Rumex japonicus* is a medicinal plant distributed in East Asia. Here, we report and characterize the complete plastid genome sequence of *R. japonicus* and size is 159,292 bp in length and contains the typical structure and gene content of other angiosperm plastomes, including two inverted repeat regions of 30,629 bp, a large single-copy region of 85,028 bp and a small single-copy region of 13,006 bp. There are 112 unique genes, including 78 protein-coding, 30 tRNAs and 4 rRNAs. We constructed a phylogenetic tree with 14 species and the phylogenetic topologies showed that *R. japonicus* was closely related to *Rheum wittrockii*.

A perennial herb plant, *Rumex japonicus* Houtt., belongs to the family Polygonaceae, is widely dispersed in the East Asia territories particularly, China, Japan and Korean Peninsula (Lee et al. 2016). It has been extensively used for the treatment of constipation, heat phlegm, jaundice, skin disease and uterine hemorrhage in traditional medicine due to presence of anthraquinones, oxanthrones, and flavonoid metabolites (Zee et al. 1998; Li et al. 2000; Zhou et al. 2005; Lee et al. 2006; Guo et al. 2011). Although, it has potential effects on antibacterial, anti-inflammatory, antioxidant and inhibitory activity against atopic dermatitis and skin disease (Zee et al. 1998; Elzaawely et al. 2005; Jang et al. 2005; Zhou et al. 2005; Lee et al. 2006; Guo et al. 2011; Xie and Yang 2014). Due to the unavailability of chloroplast (cp) information on this important medicinal plant, we sequenced and characterized the complete cp genome of *Rumex japonicus* Houtt. in the present study. The plant was collected from Dokdo island (geospatial coordinates: N37°14′20.2″, E131°52′10.6″) and the specimen stored at Yeungnam University Herbarium (YNUH), Republic of Korea (Specimen accession number: YNUH19D179). Total genomic DNA was extracted from young leaves using the Dneasy Plant Mini Kit (Qiagen) and whole-genome sequencing was performed using an Illumina HiSeq 2599 (Phyzen Ltd., South Korea).

The complete cp genome size of the plant, *Rumex japonicus* Houtt., is 159,292 bp with 37.5% of GC content which is similar to most of Polygonaceae cp genomes (GenBank accession number: MN720269). The genome has encoded two inverted repeat regions (IRa and IRb) of 30,629 bp, which is separated by one large single-copy region (LSC, 85,028 bp) and one small single-copy region (SSC, 13,006 bp). A total of 129 functional genes were identified, of which 112 were unique and included 78 protein-coding, 30 transfer RNA (tRNA) and 4 ribosomal RNA (rRNA) genes. Six protein-coding, seven tRNA and four rRNA genes were duplicated in the IR regions. This genome has encoded *rpl23* gene as a pseudogene in both IR regions. Whereas, the two intact copies of the *ycf1* gene were present in the IR region of *R. japonicus* cp genome. The similar patterns of pseudogene *rpl23* and two copies of *ycf1* were observed in other species of Polygonaceae cp genomes.

The maximum likelihood molecular phylogenetic tree was constructed using 76 protein-coding genes of 14 cp genome sequences (Figure 1). The molecular phylogenetic tree was divided into two clades, Polygonaceae species formed as one clade whereas other species of Caryophyllales formed as another clade. Interestingly, the phylogenetic topologies showed that the species *R. japonicus* is closely related to *Rheum wittrockii* with a maximum bootstrap value of 100%, whereas *R. palmatum* and *Oxyria sinensis* formed a sister clade to *R. japonicus* clade. This variation is due to presence of non-synonymous mutations in the protein-coding genes of *clpP*, *matK*, *psbB*, *rpl2*, *rpoC1*, *rpoC2* and *ycf2*. The complete plastome sequence of *R. japonicus* will provide a useful resource for the conservative genetics of this species as well as for the phylogenetic studies for Polygonaceae.

**Figure 1.**
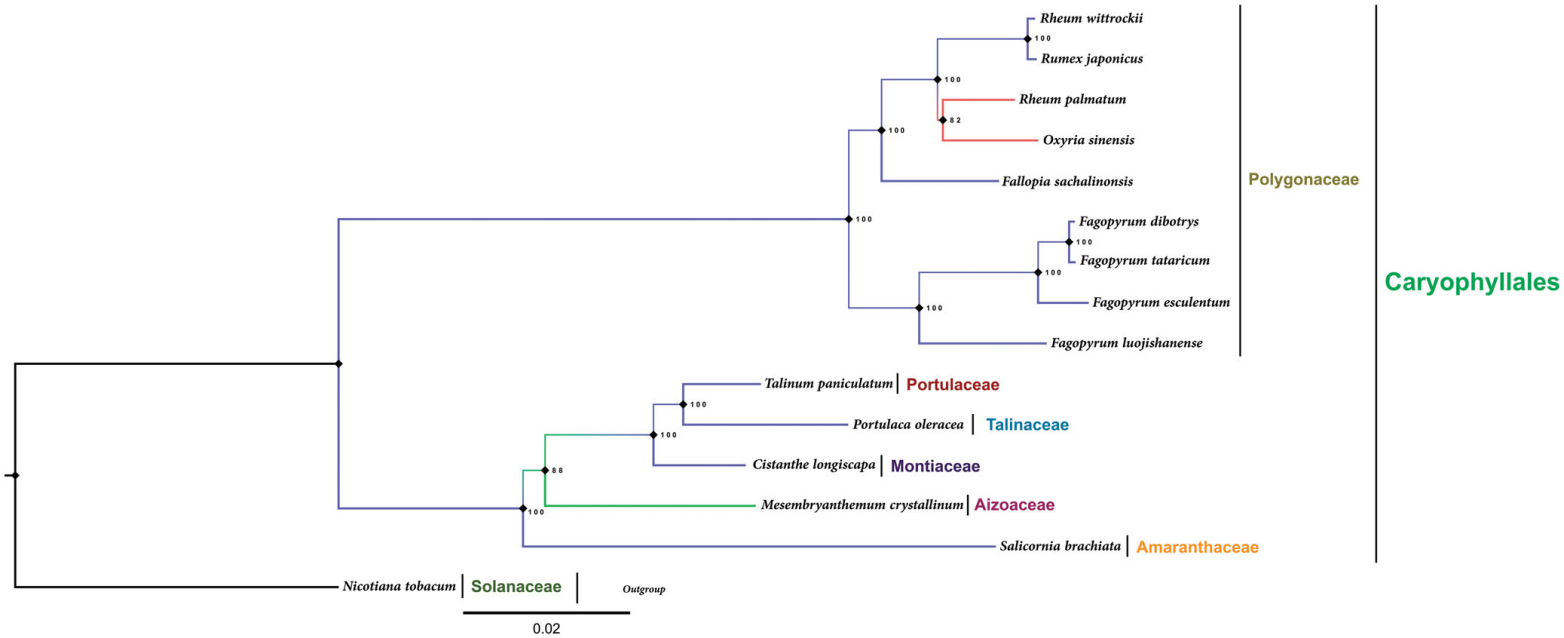
Maximum likelihood phylogenetic tree of 15 Caryophyllales species with 76 chloroplast protein-coding gene sequences. The tree was constructed by using the RAxML program and the GTR + G + I nucleotide model. The stability of each tree node was tested by bootstrap analysis with 1000 replicates. *Nicotiana tabacum* was set as the outgroup.
